# Live-cell imaging reveals the dynamics and function of single-telomere TERRA molecules in cancer cells

**DOI:** 10.1080/15476286.2018.1456300

**Published:** 2018-04-16

**Authors:** Laura Avogaro, Emmanuelle Querido, Myriam Dalachi, Michael F. Jantsch, Pascal Chartrand, Emilio Cusanelli

**Affiliations:** a Centre for Integrative Biology (CIBIO), University of Trento, Trento, Italy; b Department of Biochemistry and Molecular Medicine, Université de Montréal, QC, Canada; c Centre of Anatomy and Cell Biology, Medical University of Vienna, Vienna, Austria

**Keywords:** Telomeres, TERRA, live-cell imaging, DNA damage, cancer

## Abstract

Telomeres cap the ends of eukaryotic chromosomes, protecting them from degradation and erroneous recombination events which may lead to genome instability. Telomeres are transcribed giving rise to telomeric repeat-containing RNAs, called TERRA. The TERRA long noncoding RNAs have been proposed to play important roles in telomere biology, including heterochromatin formation and telomere length homeostasis. While TERRA RNAs are predominantly nuclear and localize at telomeres, little is known about the dynamics and function of TERRA molecules expressed from individual telomeres. Herein, we developed an assay to image endogenous TERRA molecules expressed from a single telomere in living human cancer cells. We show that single-telomere TERRA can be detected as TERRA RNA single particles which freely diffuse within the nucleus. Furthermore, TERRA molecules aggregate forming TERRA clusters. Three-dimensional size distribution and single particle tracking analyses revealed distinct sizes and dynamics for TERRA RNA single particles and clusters. Simultaneous time lapse confocal imaging of TERRA particles and telomeres showed that TERRA clusters transiently co-localize with telomeres. Finally, we used chemically modified antisense oligonucleotides to deplete TERRA molecules expressed from a single telomere. Single-telomere TERRA depletion resulted in increased DNA damage at telomeres and elsewhere in the genome. These results suggest that single-telomere TERRA transcripts participate in the maintenance of genomic integrity in human cancer cells.

## Introduction

Telomeres are nucleoprotein structures assembled at the ends of eukaryotic chromosomes which protect chromosome ends from being recognized as sites of DNA damage [1]. For this reason, telomeres are essential to genome integrity. Telomeric DNA consists of repetitive sequences (TTAGGG_n_ in vertebrates) followed by a single stranded G-rich 3′ overhang [2]. Telomeric repeats are bound by a set of telomere-binding proteins, known as the “shelterin” complex in mammalian cells, which mediate telomere function by preventing the activation of the DNA damage response. Shelterin thus functions to impede the induction of DNA repair mechanisms at chromosome ends, including non-homologous end joining (NHEJ) and homologous recombination (HR), which could lead to chromosome end fusions and genome instability [1].

Telomeres are enriched in heterochromatic marks [3,4]. Nevertheless, they are transcribed into a long noncoding RNA called TERRA (telomeric repeat-containing RNA) [5,6]. TERRA transcription starts within subtelomeric regions and proceeds towards chromosome ends, terminating within the telomeric repeat tract [7]. For this reason, TERRA molecules consist of subtelomere derived sequences at their 5′ end, and their 3′end includes arrays of *UUAGGG* repeats (reviewed in [8]). Expression of TERRA is tightly regulated by the activity of several transcription regulators, including the chromatin organizing factor CTCF [9] and the transcription factors heat shock factor 1 (HSF1) [10], Snail [11], as well as the nuclear respiratory factor NRF1 [12]. Furthermore, telomere shortening is associated with increased TERRA levels in yeast as well as human cells [13-16]. RNA fluorescence *in-situ* hybridization (FISH) and live-cell imaging analyses have shown that a subset of TERRA transcripts localizes with human telomeres [5,6,17]. At telomeres, TERRA molecules have been proposed to mediate several important functions, including regulation of heterochromatin formation [16], recruitment of chromosome end-processing and chromatin remodelling factors to dysfunctional telomeres [18,19], sustaining telomeric DNA replication [20], participating in telomere length homeostasis by regulating telomerase activity [13,14] or promoting homologous recombination among telomeres through formation of RNA-DNA heteroduplex (R-loops) at chromosome ends [21-24]. In addition to their preferential association with telomeres, recent evidence indicates that TERRA transcripts interact with numerous internal chromosomal regions to regulate widespread gene expression [25]. In line with this evidence, understanding the dynamics of TERRA molecules will be critical in order to define their function and regulation in cells. While most studies explored the cellular dynamics and function of the whole TERRA population, little is known about the impact of TERRA expressed from a single telomere on genomic integrity.

Herein, we developed a live-cell imaging assay, based on the MS2-GFP system, to visualize endogenous TERRA transcripts expressed from a single telomere in human cancer cells. This approach enabled us to investigate the spatiotemporal dynamics of single-telomere TERRA molecules and study their localization at telomeres in living cells. Depletion of TERRA transcripts expressed from a single telomere resulted in induction of DNA damage not only at telomeres but also at extratelomeric sites in the genome. Our findings provide novel insight into the dynamics and function of single-telomere TERRA molecules in human cancer cells.

## Results

### Generation of TERRA-MS2 clones in AGS human cancer cells

We previously used the MS2-GFP system to tag and image endogenous TERRA transcripts expressed from a single telomere in living yeast cells [13]. The MS2 system relies on the high affinity binding between the bacteriophage MS2 stem-loop RNA and the bacteriophage MS2 RNA binding protein and it has been widely used to study endogenous RNA molecules in living cells of various organisms, including human [26,27]. To investigate single-telomere TERRA molecules in human cancer cells, we employed the CRISPR/Cas9 genome editing tool to promote site-specific integration of a cassette containing 10×MS2 repeats and a neomycin resistance gene flanked by lox-p sites (TERRA-MS2 cassette) at subtelomere 15q in AGS cells, which is a human stomach adenocarcinoma cell line. The subtelomere 15q was chosen since expression of TERRA from human telomere 15q has been extensively validated by *in vitro* techniques [12,16,18,28]. The 15q subtelomere contains a conserved CpG rich TERRA promoter region and the TERRA transcription start sites within this subtelomere have been mapped [7,18]. Finally, the subtelomeric region of the chromosome 15q contains a unique sequence which could be targeted for the integration of the MS2-cassette (Supplementary figure S1). The AGS cells were used as a model system since TERRA expression is known to be upregulated in human stomach cancer samples [29], and the AGS cell line is a near diploid cancer cell line. This feature would favour the imaging of TERRA transcripts localization at telomeres. AGS cells are telomerase positive cells harbouring a WT p53 gene [30,31].

**Figure 1.**
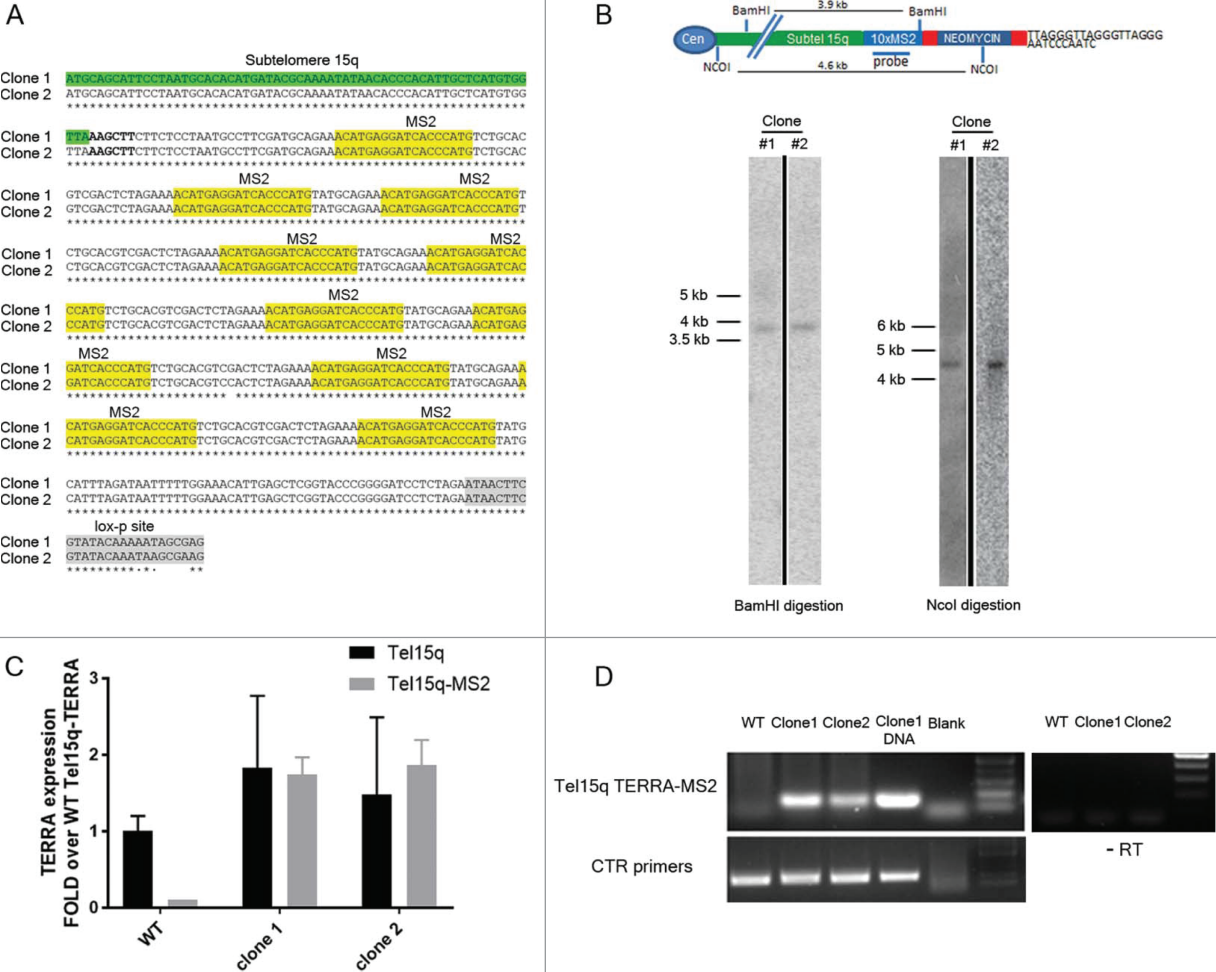
Generation and characterization of cell lines expressing MS2-tagged TERRA molecules from subtelomere 15q. (A) Sequencing analyses confirm the presence of 10×MS2 repeats at subtelomere 15q in two clones. Sequence alignment proceeds 5′ to 3′. The MS2 sequences and the loxp site present downstream of the 10xMS2 repeats are shown. (B) Southern blot screening of neomycin-resistant clones. Genomic DNA was digested with BamHI or NcoI restriction enzymes. After blotting, hybridization was performed using a radioactive MS2 sequence-specific probe. A single band of the expected size was detected in two clones. A schematic of subtelomere 15q containing the MS2 cassette is shown. Unprocessed images are shown in Figure S7. (C) RT-qPCR analyses of Tel15q-TERRA and Tel15q-TERRA-MS2 expression in WT cells and the two TERRA-MS2 clones. Data shown represent mean ± SD from two biological replicates. (D) RT-PCR analyses of Tel15q-TERRA-MS2 expression in WT cells and the two TERRA-MS2 clones. Genomic DNA extracted from clone 1 was used used as positive control.

In order to generate clones containing MS2 sequences at subtelomere 15q, AGS cells were transfected with the MS2 cassette and a vector expressing the Cas9 nickase enzyme [32,33] and guide RNAs designed to specifically promote Cas9 activity within the subtelomere 15q sequence adjacent to the telomeric repeats tract (see Figure S1 and supplementary information section). Neomycin-resistant single clones were selected and screened by PCR using primers annealing within subtelomere 15q and downstream of the MS2 repeats. DNA sequencing analyses of the PCR fragments confirmed the presence of 10×MS2 sequences (Figure 1A). In order to confirm the single integration of the MS2 cassette within subtelomere 15q, neomycin-resistant single clones were further screened by Southern blot using a MS2 sequence-specific probe. In two different clones, digestion of genomic DNA using NcoI or BamHI restriction enzymes resulted in the detection of a single band of 4.6 kb (NcoI digestion) or 3.9 kb (BamHI digestion) in size, indicating specific integration of the MS2 cassette within subtelomere 15q (Figure 1B). These two clones (thereafter named TERRA-MS2 clone 1 and 2), were infected with a Cre-GFP expressing adenovirus in order to remove the neomycin resistance gene, leaving only MS2 sequences within subtelomere 15q. RT-qPCR analyses confirmed the expression of Tel15q-TERRA-MS2 transcripts in both TERRA-MS2 clones (Figure 1C). Tel15q-TERRA-MS2 expression levels were comparable to the Tel15q-TERRA levels detected in WT cells. Expression analyses of TERRA from multiple telomeres also showed comparable levels between TERRA-MS2 clones and WT cells (Figure S2). The expression of Tel15q-TERRA-MS2 transcripts in TERRA-MS2 clones was further analyzed by RT-PCR using an RT primer annealing on the telomeric repeat tract and PCR primers annealing at the 5′ of the integration site, within the subtelomere 15q sequence, and downstream of the 10×MS2 sequences. Detection of an approximately 500 bp sized PCR band confirmed the expression of 10×MS2-tagged TERRA transcripts from subtelomere 15q in both clones (Figure 1D). Overall these results show that the two different TERRA-MS2 clones selected in the AGS cell line contain 10×MS2 sequences at subtelomere 15q and express Tel15q-TERRA-MS2 transcripts.

### Live-cell imaging analyses of TERRA-MS2 clones

**Figure 2.**
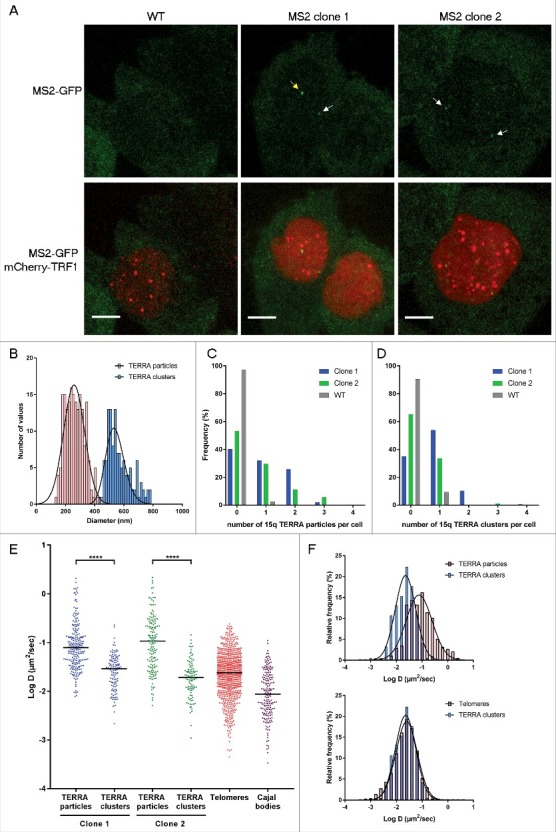
Imaging Tel15q TERRA-MS2 molecules in living AGS cells. (A) Images of AGS WT (control), clone 1 and clone 2 cells expressing MS2-GFP. Tel15q TERRA-MS2 foci are indicated by arrows (yellow for cluster and white for particles). Scale bar: 5 µm. (B) Two populations of Tel15q TERRA-MS2 foci are detected in AGS clones. (C) Expression of Tel15q TERRA-MS2 particles in AGS WT, clone 1 and clone 2 cells (N = 88 to 157 cells). (D) Expression of Tel15q TERRA-MS2 clusters in AGS WT, clone 1 and clone 2 cells (N = 95 to 154 cells). (E) Diffusion coefficients (in log D) of Tel15q TERRA-MS2 particles and clusters from clones 1 and 2. Diffusion coefficients of telomeres (TRF1-mCherry) and Cajal bodies (Coilin-mCherry) are included for comparison. (F) Overlay between the frequency distributions of Tel15q TERRA-MS2 clusters, particles and telomeres diffusion coefficients (log D).

In order to image Tel15q-TERRA-MS2 transcripts in live cells, AGS WT and TERRA-MS2 clones were infected with a lentivirus expressing a superfolder GFP-fused MS2 RNA-binding protein which specifically recognizes MS2 RNA sequences. Live-cell confocal imaging revealed the formation of TERRA-MS2-GFP foci in both TERRA-MS2 clones, while no foci were detected in AGS WT cells (Figure 2A). TERRA-MS2-GFP foci were predominantly detected within the nucleus (Figure S3). In order to gain insight into the dynamics of TERRA transcripts, we performed single particle tracking to analyze the kinetics of TERRA-MS2-GFP foci in living cells. Interestingly, by measuring the size distribution of TERRA foci, we detected two different populations of TERRA-MS2-GFP foci: small TERRA particles, with an average diameter of 250 nm, and large TERRA clusters, showing an average diameter of 550 nm (Figure 2B). Quantification analyses of movies acquired by streaming indicate that 47% (clone 2) to 60% (clone 1) of AGS cells express Tel15q TERRA small particles (Figure 2C). Using 3D acquisition to image the whole nucleus, Tel15q TERRA large particles were detected in 35% of cells from clone 2 and up to 65% of cells from clone 1 (Figure 2D). Although the number of Tel15q TERRA-MS2 particles per cell is low, these results show that this lncRNA is expressed in a large proportion of the cell population.

Measures of diffusion coefficient (*D*) revealed that Tel15q TERRA single particles move faster than TERRA clusters. Tel15q TERRA single particles display *D* ranging between 2.1 and 5 × 10^−3^µm^2^/sec (mean *D =* 0.09 µm^2^/sec; Figure 2E), which is consistent with the diffusion coefficient of previously imaged TERRA molecules (mean *D* = 0.15 µm^2^/sec) [17]. However, Tel15q TERRA clusters display *D* between 0.15 and 1 × 10^−3^ µm^2^/sec (mean *D* = 0.02 µm^2^/sec), with similar diffusion coefficients observed in both clones (Figure 2E). Compared to previously imaged nuclear mRNAs [34,35], the size and dynamics of TERRA foci suggest that the small particles correspond to single Tel15q TERRA molecules, while the large foci represent clusters of several Tel15q TERRA molecules (supplementary movie 1 and 2).

**Figure 3.**
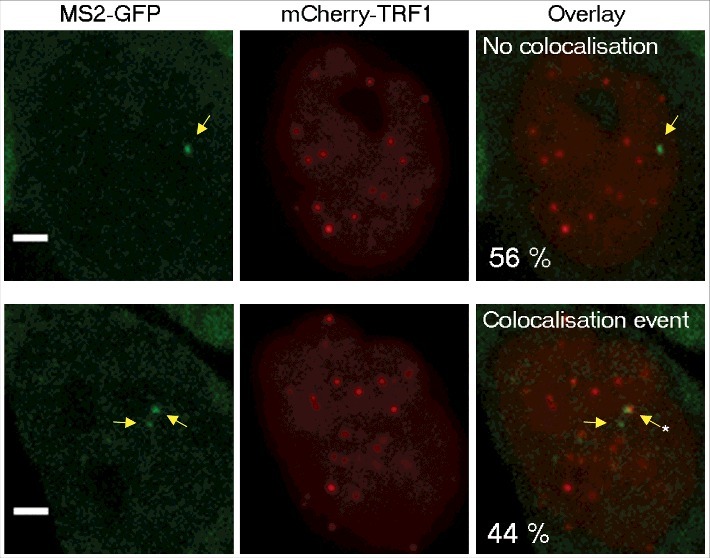
Colocalization between Tel15q TERRA clusters and telomeres. Quantification of colocalization events between TRF1-mCherry labelled telomeres and Tel15q TERRA-MS2 clusters observed during time-lapse imaging. TERRA-MS2 clusters are indicated by yellow arrows. Asterisk marks the co-localization event. Representative images of MS2-GFP and TRF1-mCherry signals co-localizing (bottom) or not (top) are shown. Frequency of occurrence is indicated as percentage (N = 55 cells). Scale bar: 5 µm.

For comparison, we analyzed the dynamics of telomeres and Cajal bodies by expressing mCherry-fused TRF1 (a telomere-binding protein and shelterin complex component) or Coilin (a component of Cajal bodies) in these cells. Interestingly, the distribution of diffusion coefficients of TERRA clusters clearly overlaps with the distribution of telomeres diffusion coefficient values (Figure 2F), raising the possibility that TERRA clusters may be associated with telomeres. To explore this possibility, we expressed a mCherry-fused TRF1 protein in TERRA-MS2 clones in order to simultaneously image telomeres and Tel15q TERRA-MS2-GFP clusters. Time-lapse imaging was performed using z-stack and simultaneous two colours image acquisitions to detect Tel15q TERRA-telomeres interactions. In these conditions, only Tel15q TERRA clusters can be detected. Interestingly, we observed co-localization events between TERRA-MS2 clusters and telomeres in 40% of the cells (Figure 3). The co-localizations were transient, as they were detected in only one or two time points per experiment (30–60 seconds). Based on these findings, we conclude that although their diffusion coefficients overlap with the diffusion coefficients of telomeres in AGS cells, TERRA clusters only transiently localize at chromosome ends.

### Single-telomere TERRA depletion results in activation of the DNA damage response

**Figure 4.**
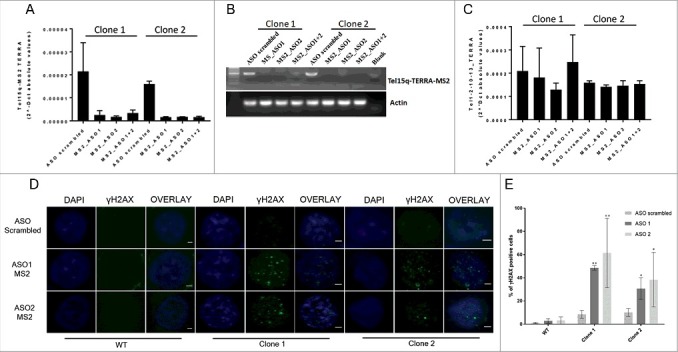
Validation of Tel15q MS2-TERRA depletion upon transfection of antisense oligonucleotides (ASO) in AGS clones and analyses of DNA damage by immunofluorescence experiments. (A) RT-qPCR analysis of Tel15q TERRA-MS2 expression in the two MS2 clones. RNA was extracted from cells upon 48 hours from transfection of ASO-MS2 or ASO scrambled. Values shown were normalized with actin. N = 2. (B) RT-PCR analysis of Tel15q TERRA-MS2 expression in the two MS2 clones upon transfection of the ASO, as in A. Upon RT reaction, a specific set of primers was used for PCR reaction to detect a 500 bp long fragment which includes the 10xMS2 repeats. (C) RT-qPCR analyses of Te1-2-10-13q TERRA in the two MS2 clones upon transfection of ASO, as in A. (D) TERRA-MS2 clones and AGS WT cells were transfected with MS2 antisense oligonucleotides (ASO) or scrambled ASO as negative control. Immunofluorescence experiments were performed using γH2AX antibody to analyse DNA damage. Nuclei were stained by DAPI. Representative images for each transfection are shown. Scale bar: 1 µm (E) Quantification of the number of γH2AX-positive cells upon depletion of TERRA-MS2 transcripts. At least 300 cells were analysed in two different experiments. **: *p*-value< 0.0002; *: *p*-value < 0.003. *P*-values were calculated using an unpaired t-test.

In order to get insight into the function of TERRA molecules expressed from a single telomere, we used antisense oligonucleotides (ASO) to deplete Tel15q TERRA. We designed two different chemically-modified (phosphorothioate) locked-nucleic acid (LNA) ASO designed to specifically recognize and promote RNase-H degradation of MS2 RNA sequences (MS2-ASO) to deplete single-telomere TERRA transcripts in TERRA-MS2 clones. Transfection of the TERRA-MS2 clones with either of the two MS2-ASO resulted in downregulation of TERRA-MS2 RNA expression, as compared to transfection with a scrambled-ASO, used as negative control, as detected by RT-qPCR (Figure 4A) and RT-PCR analyses (Figure 4B). No changes in the expression of TERRA from other telomeres were observed upon MS2-ASO transfection (Figure 4C), indicating that the MS2-ASO specifically target the Tel15q TERRA-MS2 transcripts. Since TERRA is involved in the maintenance of genomic integrity [18,36] we explored the impact of Tel15q TERRA-MS2 depletion on the accumulation of DNA damage. Interestingly, depletion of MS2-tagged TERRA transcripts in the two AGS clones resulted in increased number of cells showing multiple γH2AX foci within the nucleus as detected by immunofluorescence experiments using an antibody specific to γH2AX, a marker of DNA damage (Figure 4D and E). Importantly, transfection of WT cells with the same two MS2-ASO did not induce DNA damage foci in these cells (Figure 4D and E), showing that the effect of these ASO is specific toward the TERRA-MS2 RNA. Control experiments confirmed the specificity of the γH2AX signal (Figure S4 and S5). These results indicate that depletion of TERRA from a single telomere associates with increased DNA damage in cells.

**Figure 5.**
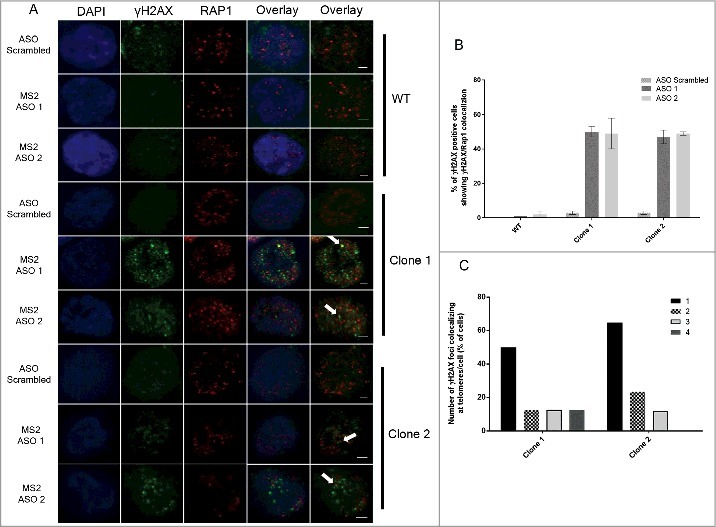
Immunofluorescence experiments show increase in γH2AX foci co-localizing with telomeres upon depletion of TERRA-MS2 transcripts. (A) AGS WT and TERRA MS2 clones were transfected with ASO scrambled or MS2-specific ASO. Immunofluorescence experiments were performed using γH2AX and Rap1 specific antibodies. The co-localization between γH2AX and Rap1 were analysed by two colours Z-stack imaging. γH2AX foci are in green, Rap1 foci are in red. White arrows indicate representative co-localization events detected. Nuclei were stained by DAPI. (B) Quantification of colocalization occurrence between γH2AX foci and Rap1 signals. Results are shown as percentage of γH2AX-positive cells in which colocalization between γH2AX foci and Rap1 signals was detected. N = 2. (C) Quantification of the number of γH2AX foci colocalizing with Rap1 foci per cell. The Y axis shows percentage of cells where the indicated number of γH2AX foci colocalizing with Rap1 foci were detected.

We then investigated whether the DNA damage induced by Tel15q TERRA-MS2 depletion occurs at telomeres. With this aim, we performed double immunofluorescence experiments using antibodies specific to γH2AX and Rap1, a telomere-binding protein and component of the shelterin complex (Figure 5A) [37]. Interestingly, quantification analyses revealed a significant increase in the number of cells showing DNA damage signal co-localizing with telomeres in both TERRA-MS2 clones (Figure 5B). In most cases, γH2AX signal colocalized with only a single telomere (Figure 5C) and most DNA damage foci were not at telomeres (Figure 5A). Similar results were obtained by performing immunofluorescence experiments using γH2AX antibody followed by DNA-FISH using a fluorescent-labelled telomeric repeat-specific probe to detect telomeres in fixed cells (Figure S6). Overall these results indicate that depletion of TERRA from a single telomere results in DNA damage detected not only at telomeres but also elsewhere in the genome, suggesting that TERRA transcripts participate in the maintenance of genomic integrity in cancer cells.

## Discussion

In this study, we developed a live-cell imaging assay to image MS2-tagged TERRA molecules expressed from a single telomere in human cancer cells. We report that in human AGS cancer cells, MS2-tagged TERRA transcripts expressed from telomere 15q form discrete foci within the nucleus. Three-dimensional size distribution and single particle tracking analyses revealed the formation of two populations of nuclear TERRA-MS2-GFP foci, distinguishable by their sizes and diffusion coefficients. In particular, we detected small TERRA particles, with a diffraction-limited diameter and diffusion coefficients similar to previously reported single TERRA RNA molecules [17]. In addition, we observed larger TERRA foci, showing slower dynamics within the nucleus. Considering their sizes and kinetics, larger TERRA foci could represent clusters of TERRA transcripts which may be bound to chromatin or assembled in large ribonucleoprotein particles (RBPs). Interestingly, similar TERRA clusters have been observed in budding yeast [13], suggesting that the capacity of this lncRNA to form high-ordered structures may be conserved among eukaryotes.

Simultaneous imaging of both Tel15q TERRA-MS2 and telomeres revealed colocalization events between TERRA clusters and telomeric foci. However, single-telomere TERRA transcripts only transiently localize with telomeres, which is consistent with previous studies showing that only a small fraction of total TERRA transcripts co-localize with telomeres in fixed or living cells [5,17]. A transient localization of single-telomere TERRA molecules at chromosome ends was also previously observed in yeast by live-cell imaging, indicating that this is a conserved feature of TERRA transcripts [13]. By performing single particle tracking analyses, we observed that the diffusion coefficients of TERRA clusters overlap with the diffusion coefficients of telomeres. Still, a significant fraction of Tel15q TERRA-MS2 small particles also display the same dynamics as telomeres, suggesting that single TERRA molecules may also interact with telomeres or with other sites on the chromatin.

We then used chemically-modified antisense oligonucleotides to deplete MS2-tagged Tel15q TERRA molecules in our AGS clones. Unlike other studies in which total TERRA was depleted using ASO or siRNAs [25,38], the presence of MS2 repeats at Tel15q in our AGS clones, and not in WT cells, allows a specific depletion of Tel15q TERRA-MS2. Interestingly, depletion of Tel15q TERRA-MS2 transcripts resulted in the induction of DNA damage in both AGS clones, as detected by an increased number of cells with γH2AX foci. Furthermore, we observed a significant increase in the number of γH2AX foci colocalizing with one or a few telomeres upon Tel15q TERRA-MS2 depletion. In future experiments, it will be important to investigate whether depletion of TERRA-MS2 transcripts results in DNA damage at the MS2-tagged telomere. Indeed, recent evidence indicates that TERRA expression facilitates replication *in cis* of the TERRA transcribing telomere [20], thus single-telomere TERRA downregulation may perturb DNA replication at the TERRA-depleted telomere, resulting in DNA damage.

However, the majority of γH2AX foci detected upon Tel15q TERRA-MS2 depletion are not localized at telomeres, suggesting that Tel15q TERRA-MS2 may also act on extratelomeric sites. In mouse ES cells, TERRA molecules have been recently shown to bind chromatin throughout the genome [25]. We hypothesize that the DNA damage induced upon TERRA-MS2 depletion may result from altered genomic integrity at chromatin sites normally bound by TERRA. It is tempting to speculate that distinct single-telomere TERRA transcripts preferentially act *in cis*, regulating telomere function and gene expression at the chromosome from which they originate. A similar behaviour is well known for other *cis*-acting long noncoding RNAs such as Xist [39]. This new system to study and deplete single-telomere TERRA molecules will provide a significant advantage to explore the function and regulation of TERRA in cancer cells.

## Materials and methods

### Cell lines and culturing conditions

AGS cells were a kind gift from Christian Baron (Université de Montréal). Cells were grown in Ham's F12K medium (Thermo Scientific, 21127030) supplemented with glutamine, Pen-strep, and 10% FBS. For TERRA-MS2 clone selection, cells were grown in F12K medium containing Neomycin 0.4 ug/ml. Upon elimination of the Neomycin gene, by Cre expression, cells were cultured in complete F12K medium. Cells were regularly tested for mycoplasms. For details on TERRA-MS2 clone selection please see supplemental online material.

### Southern blot

Cells were lysed in 1ml lysis buffer (10 mM tris pH 7.4, 10 mM EDTA pH8, 10 mM NaCl, 0.5% SDS) containing RNAse A, and incubated at 37°C for 1 hour. Then proteinase K (Thermo Scientific, EO0492) was added to 0.2 mg/ml final concentration, and incubated overnight at 37°C. Genomic DNA was extracted by phenol/chloroform extraction and precipitated using ethanol 100% in the presence of Sodium Acetate. DNA pellets were resuspended in water. 5–10 micrograms of DNA were digested overnight with BamHI or NcoI restriction enzymes. Digested DNA was run on 0.8% agarose gel overnight 35 Volts in TAE (Tris/Acetic Acid/EDTA) buffer. After run, gel was stained with ethidium bromide for 30 minutes and imaged on Chemidoc XRS+ (Biorad) instrument. The gel was then incubated in denaturating solution (0.5 M NaOH, 1 M NaCl) for 45 minutes and in neutralization solution (1.5 M Tris pH 7.5) for 1 hour at room temperature. Blotting on a positively charged nylon membrane (GE Healthcare, RPN203B) was performed by capillary transfer using 10xSSC buffer (0.15M tri-sodium citrate, 1.5M sodium chloride) as transfer solution, overnight at room temperature. DNA was crosslinked on the membrane by UV irradiation and membrane blocked in hybridization buffer (10% dextran sulfate, 6xSSC, 1%SDS). Hybridization was performed using a radioactively labelled MS2-sequence specific probe (PCR product containing 10xMS2 sequence) by overnight incubation at 65°C in hybridization buffer. Images were acquired using a Typhoon Biomolecular Imager (GE healthcare).

### RT-qPCR and RT-PCR analyses

Total RNA was extracted from cells grown in 6 well plates or 10cm dishes using Trizol reagent (Thermo Scientific, 15596026) according to manufacturer instructions. 3 µg of RNA was treated with DNaseI (Thermo Scientific, 89836) then 300 ng of DNase-treated RNA was retrotranscribed using SuperScript III reverse transcriptase (Thermo Scientific, 18080044) at 42°C for 1h. For TERRA, a C-rich primer was used for retrotranscription (RT) reaction (primer sequence: CCCTAACCCTAACCCTAACCCTAACCCTAA) while a specific primer was used for actin RT reaction (primer sequence: AGTCCGCCTAGAAGCATTTG). A total of 2 µl of RT reaction was used for qPCR or PCR reaction. qPCR reactions were performed in duplicates using Kapa qPCR Sygreen Mastermix (PCRBIOSYSTEMS, PB20.14-05) on a Biorad CFX96 sequence detection system. Analyses were performed by calculating the averages and SDs of the 2^−Dct^ values, normalized on actin mRNA, for each duplicate. PCR reactions were performed using a Pfu DNA polymerase (Thermo Scientific, EP0502). qPCR and PCR primer sequences are shown in supplementary table 1.

### Live cell imaging

AGS cell lines were infected with lentiviral vectors expressing MS2-GFP or mCherry-TRF1 fusion proteins (mCherry-Coilin was also used for some images). For imaging, the AGS cells were plated in 35 mm glass-bottom dishes (FluoroDish FD35-100, World Precision Instruments) in DME w/o phenol red supplemented with glutamine, Pen-strep, 10% FBS and 25 mM HEPES pH 7.4. Images were acquired on a Zeiss Axio-Observer Z1 spinning disk confocal microscope equipped with two Evolve EMCCD cameras (Photometrics, AZ, USA) for simultaneous imaging of GFP and RFP. The images were captured with a 100x/1,46 alpha Plan Apochromat DIC M27 objective in an imaging chamber maintained at 37°C with 5% CO2. For TERRA particle tracking and expression counts, the cells were filmed in streaming at 100 ms interval. For TERRA-TRF1 colocalisation analysis and TERRA cluster expression counts, GFP and mCherry image Z-stacks were acquired at 30 second intervals. Displacements of the TERRA RNA clusters and single particles were tracked with the ImageJ TrackMate plugin [40]. Tracks containing five or more points were selected for diffusion coefficient analysis as described in [35]. For quantification of nuclear versus cytoplasmic TERRA clusters, DNA labelling was performed using the live-cell fluorigenic probe Sir-DNA (Spirochrome, SC007) according to manufacturer instructions.

### ASO sequences and transfection protocol

The antisense oligonucleotides “GapmeRs” used in this study were custom designed by Exiqon (Exiqon A/S, Denmark) to specifically target and promote RNase H degradation of MS2 RNA sequences. A scrambled ASO was acquired by Exiqon as negative control. All ASO were synthesized by Exiqon using a phosphorotioate chemistry and including locked nucleic acid (LNA) bases. Scrambled ASO sequence: AACACGTCTATACGC; MS2_ASO1 sequence: GCATCGAAGGCATTAG; MS2_ASO2 sequence: TTCTAGAGTCGACGTG. For transfections, cells were transfected at a 60% confluence using JetPRIME transfection reagent (PolyPlus, cat.n. 101–40 N) according to manufacturer instructions. Briefly, a mix containing the MS2-ASO or scrambled ASO at a final concentration of 25 nM and 4 µl of transfection reagent was added to the cells seeded 36 hours in advance in 6 wells plates, each well containing 2ml of complete culturing medium. The following day, medium was changed and immunofluorescence experiments or TERRA expression analyses were performed after 48 hours from transfection.

### Immunofluorescence

Cells were grown on glass coverslips. Where indicated, treatment with neocarzinostatin (SIGMA, N9162) was performed by incubating the cells in complete medium containing neocarzinostatin to 50ng/ml final concentration for 1 hour at 37°C. After two washes in 1xPBS (5 minutes at room temperature each) cells were fixed in 4% paraformaldehyde/1xPBS solution for 10 minutes at room temperature. After three washes in 1xPBS, 5 minutes at room temperature each, cells were permeabilized with 0.2% Triton in 1xPBS for 10 minutes at room temperature. Cells were then washed three times in 1xPBS for 5 minutes at room temperature and a blocking step was performed for 1 hour at room temperature in PBG buffer, containing 0.5% BSA (SIGMA, A7030-50G) and 0.2% gelatin from cold water fish skin (SIGMA, G7765) dissolved in 1xPBS. Incubation with primary antibodies was performed for 1 hour at room temperature. For γH2AX detection, anti-phospho Histone H2AX (Ser 139) antibody (SIGMA, H139) was used at 1:500 dilution in PBG buffer; for Rap1 detection, anti-TERF2IP antibody (Novus Biologicals, NB100292) was used (1:500 dilution). After antibody incubation, coverslips were washed three times in a 1xPBS solution containing 1% BSA for 5 minutes at room temperature, then incubated with secondary antibodies for 1 hour at room temperature. The secondary antibodies used were goat anti-Mouse IgG Alexa Fluor Plus 488 (Thermo Scientific, A32723) (1:1000 dilution in PBG buffer); goat anti-Rabbit IgG Alexa Fluor 647 (Thermo Scientific, A21245) (1:500 dilution in PBG buffer). After two washes in 1xPBS solution containing 1% BSA, 5 minutes at room temperature each, and two washes in 1xPBS, 5 minutes at room temperature each, nuclei were stained by incubating coverslips in 1xPBS solution containing 1 ng/ml DAPI (Thermo Scientific, D1306) for 5 minutes at room temperature. Coverslips were mounted on slides using prolong diamond antifade mountant reagent (Thermo Scientific, P36965). Coverslips were then imaged using a Nikon CrestOptics X light spinning disc confocal microscope (CrestOptics) equipped with a PRIME sCMOS camera (Photometrix). The images were captured with a 60x/1.4 Plan Apochromatic objective.

### Immunofluorescence/DNA-FISH

Cells grown on coverslips were treated as indicated above for immunofluorescence analyses. After primary and secondary antibody incubations, coverslips were fixed in 4% paraformaldehyde/1xPBS solution for 10 minutes at room temperature, washed 3 times in 1xPBS for 5 minutes at room temperature and dehydrated in ethanol series, 75%, 85% and 100% ethanol for 5 minutes each and allowed to air dry at room temperature. A TAMRA-labelled telomeric repeat-specific LNA probe (/56TAMN/CCCTA+AC+CCT+A+AC+CCTAACCCT+A+ACCCT+A+ACCCT+AA/36TAMTSp/, Exiqon A/S, Denmark) was added to coverslips in a buffer containing 70% formamide, 10 mM Tris ph7.4, 1 mg/ml blocking solution (Roche, 11096176001) and the coverslips were denatured on a heat block (10 minutes at 80°C) and incubated 2 hours in the dark at 30°C. The coverslips were washed twice with 70% formamide, 10 mM Tris-HCl pH7.4 for 15 minutes each and three times with 1xPBS for 5 minutes each. Coverslips were then DAPI stained and mounted on slides as described in immunofluorescence protocol. Images were acquired with a ZEISS Axio Observer Z1 microscope equipped with ApoTome module using a Plan Apochromat 63x/1.4 objective.

### RNA-FISH analyses

Cells were grown on glass coverslips, washed once in 1xPBS, incubated 7 minutes in ice-cold CSK buffer (100 mM NaCl, 300 mM Sucrose, 3 mM MgCl_2_,10 mM PIPES, 0.5% Triton X-100), rinsed in 1xPBS and fixed in PBS pH7 solution containing 4% formaldehyde for 10 minutes at room temperature. Coverslips were then rinsed in Et-OH 70% and dehydrated through alcohol series (70%, 85% and then 100% for 5 minutes each at room temperature). Coverslips were subsequently air dried. At this point, when indicated, RNase A treatment was performed by incubating the coverslips in RNase A solution 1 mg/ml at 37°C for 1h. For total TERRA RNA detection, hybridization was performed using either a telomeric PNA probe, TelC-FAM, (PANAGENE, F1001) or a TAMRA-labelled telomeric LNA probe (LNA sequence: CCCTAaCcCTaaCcCTAACCCTaaCCCTaaCCCTaA) (Exiqon A/S, Denmark), 5ng per coverslip incubated for 1 hour at 37°C in 20 µl hybridization buffer (2X SSC, 10% dextran sulfate, 2 mg/ml BSA, 20 mM VRC). Coverslips were then washed three times in 0.1xSSC, 5 minutes at 60°C each, once in 2xSSC buffer for 5 minutes at room temperature and subsequently dehydrated through alcohol series as described above. After DAPI staining (1ng/ml DAPI solution in 1xPBS for 5 minutes at room temperature) coverslips were mounted on slides using the prolong diamond antifade mountant reagent (Thermo Scientific, P36965). Coverslips were then imaged using a Nikon CrestOptics X light spinning disc confocal microscope (CrestOptics) equipped with a PRIME sCMOS camera (Photometrix). The images were captured with a 60x/1.4 Plan Apochromatic objective.

### Statistical analyses

GraphPad Prism 7 (GraphPad) was used for statistical analyses. P-values were evaluated by unpaired t-test.

The authors report no conflict of interest.

## Supplementary Material

Suppl_mate_Live-cell_imaging_reveals_the_dynamics_and_function_of_single-telomere_TERRA.zip
