# Supplementary material for: Live-cell imaging reveals the dynamics and function of single-telomere TERRA molecules in cancer cells
Source: RNA Biol. 2018 Apr 16;15(6):787–96. doi: 10.1080/15476286.2018.1456300 (PMC6152429; doi:10.1080/15476286.2018.1456300)
Supplement: Suppl_mate_Live-cell_imaging_reveals_the_dynamics_and_function_of_single-telomere_TERRA.zip [file krnb-15-06-1456300-s001.zip › Supplementary Text.docx]

**Cloning of the Tel15q-TERRA-MS2 cassette:**

The MS2 cassette consists of 800nt long subtelomeric 15q sequence, followed at its 3’ by 10xMS2 repeats, a Neomycin resistance gene flanked by lox-p sites and terminating with a 300nt long telomeric repeat tract. The cassette was cloned in pBlue Script vector. Briefly, the Neomycin gene was PCR amplified using primers containing lox-p sites and cloned in pUC18 vector, generating pUC-LoxpNEO vector. 10xMS2 repeats were PCR amplified from 3’TLC1-10xMS2 pUG6 vector (Cusanelli et al., 2013) and cloned SacI in pUC-loxpNEO vector, at the 5’ of the NEO gene. The sequence 10xMS2-loxp-NEO was PCR amplified from pUC18-MS2-loxp-NEO vector and cloned HindIII/EcoRI in pBS II SK vector. Telomeric repeat sequences were obtained from pSX-Neo-1.6_T2AG3 vector by NotI-EcoRI restriction enzymes digestion and cloned NotI/EcoRI in pBS MS2loxpNEO vector. Then human subtelomeric sequence adjacent to the telomeric repeat tract was PCR amplified from the FOSMID clone ABC10-44089300D10 (Thermo Scientific) and cloned XhoI/HindIII in pBS-MS2loxpNEO vector, generating the final MS2 cassette. The CRISPR/Cas9 genome editing tool was used to promote site-directed recombination of the MS2 cassette at subtelomere 15q of AGS cells. For this reason, in order to reduce the activity of the Cas9 enzyme on the integrated cassette, the PAM sites recognised by the guide RNAs used to promote homologous recombination were mutated on the MS2 cassette. To this aim, single nucleotide substitutions were generated within each of two PAM sites by site directed mutagenesis. The final MS2 cassette containing mutated PAM sites was verified by DNA sequencing. Before transfection, the cassette was removed from pBS vector by XhoI/NotI digestion and transfected as a linearized fragment.

**Generation of TERRA-MS2 clones in AGS cells:**

AGS cells were transfected in 6 well plates using the Jet PRIME transfection reagent (Polyplus, cat.n. 101-40N) following manufacturer instructions. For each well, the DNA mix transfected contained 100ng of pX335 vector expressing the Cas9 nickase enzyme and a short guide RNA specific for the subtelomere 15q sequence and 1 µg of the MS2 cassette. Two different guide RNAs targeting subtelomere 15 sequence were cloned in pX335 vector using BbsI restriction site, generating two different sgRNA-pX335 vectors. A mix of these two vectors (50ng each) was used for the transfections.

After one day from transfection, cells were trypsinized and transferred to a 10cm dish containing G418 selection medium at the final concentration of 0.7µg/ml of F12K medium. After 10 days’ selection, single clones were picked and transferred in 96 well plates. Single clones were screened by PCR, DNA sequencing and southern blot, as indicated in text. Two different positive clones were identified, out of about one hundred clones screened. The Neo gene was removed from the integrated cassette by infecting the clones with a Cre-GFP expressing adenovirus (a kind gift from Dr. Emilio Casanova). Removal of the cassette was confirmed by negative selection in G418 containing medium.

**Table1. List of the primers used in this study, sequences are 5’ to 3’**

| Primer name | Primer sequence | Application |
| --- | --- | --- |
| Tel15q-S1 | GCAGCGAGATTCTCCCAAGC | Sense primer used to detect Tel15q and Tel15q-MS2 TERRA expression by qPCR and PCR |
| hTel15q-AS | TAACCACATGAGCAATGTGGGTG | Antisense primer used to detect Tel15q and Tel15q-MS2 TERRA expression by qPCR |
| Tel15q-MS2-AS | ATGTTTCTGCATCGAAGGCATTAGG | Antisense primer used to detect Tel15q-MS2 TERRA expression by qPCR |
| MS2-3’-AS | GGGTACCGAGCTCAATGT | Antisense primer used to detect Tel15q-MS2-TERRA expression by PCR |
| Tel1-2-10-13q-S | GAATCCTGCGCACCGAGAT | Sense primer used to detect TERRA expression from multiple telomeres by qPCR |
| Tel1-2-10-13q-AS | CTGCACTTGAACCCTGCAATAC | antisense primer used to detect TERRA expression from multiple telomeres by qPCR |

**Supplementary Reference:**

Beerman TA, Goldberg IH. DNA strand scission by the antitumor protein neocarzinostatin. Biochem. Biophys. Res. Commun. 1974 Aug;59(4):1254-1261. [doi: 10.1016/0006-291X(74)90449-5](https://doi.org/10.1016/0006-291X(74)90449-5). PubMed PMID: 4369938
