# Supplementary material for: Live-cell imaging reveals the dynamics and function of single-telomere TERRA molecules in cancer cells
Source: RNA Biol. 2018 Apr 16;15(6):787–96. doi: 10.1080/15476286.2018.1456300 (PMC6152429; doi:10.1080/15476286.2018.1456300)
Supplement: Suppl_mate_Live-cell_imaging_reveals_the_dynamics_and_function_of_single-telomere_TERRA.zip [file krnb-15-06-1456300-s001.zip › Supplementary Figures-revised.pptx]

## Slide 1
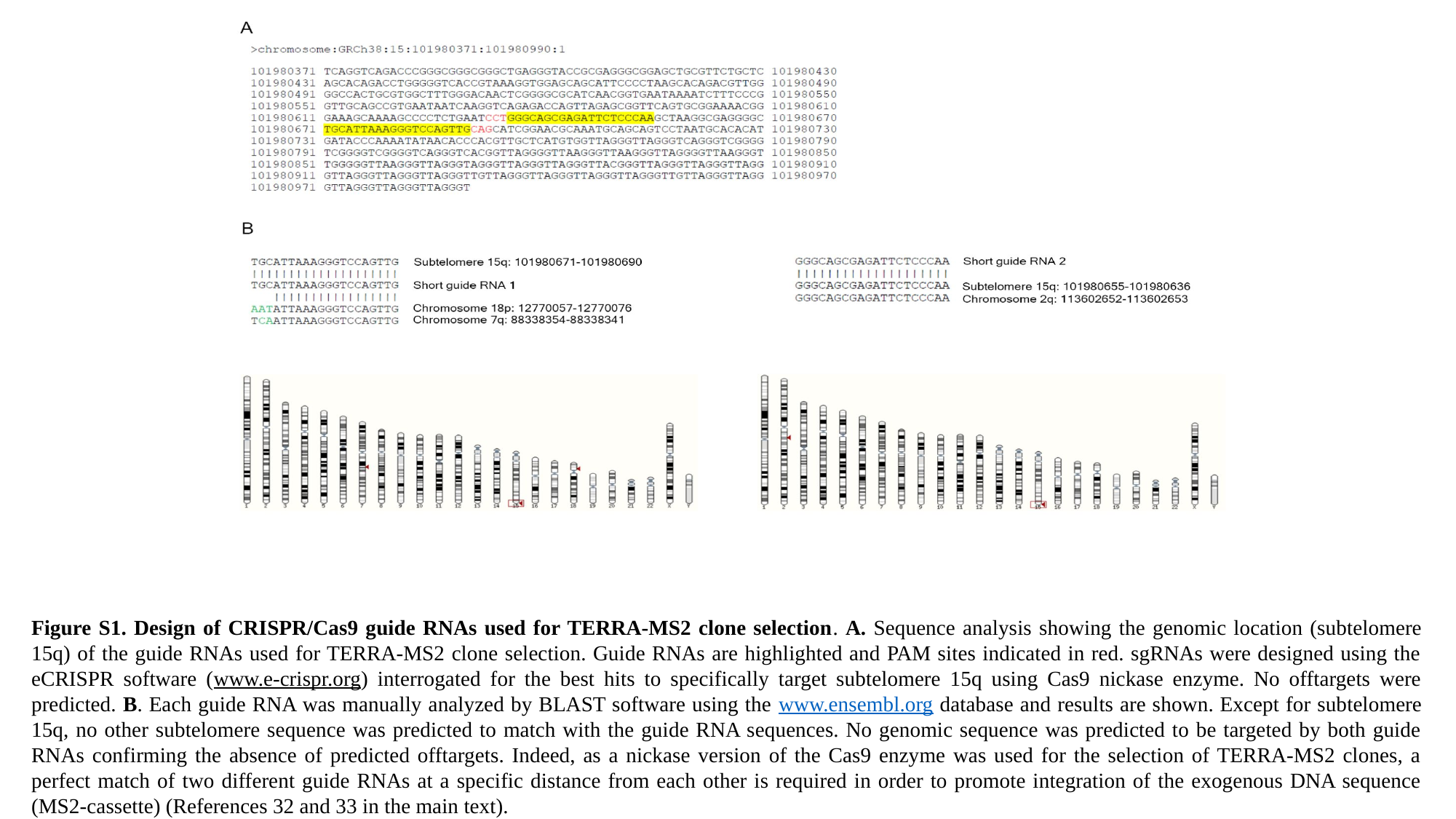

Figure S1. Design of CRISPR/Cas9 guide RNAs used for TERRA-MS2 clone selection. A. Sequence analysis showing the genomic location (subtelomere 15q) of the guide RNAs used for TERRA-MS2 clone selection. Guide RNAs are highlighted and PAM sites indicated in red. sgRNAs were designed using the eCRISPR software (www.e-crispr.org) interrogated for the best hits to specifically target subtelomere 15q using Cas9 nickase enzyme. No offtargets were predicted. B. Each guide RNA was manually analyzed by BLAST software using the www.ensembl.org database and results are shown. Except for subtelomere 15q, no other subtelomere sequence was predicted to match with the guide RNA sequences. No genomic sequence was predicted to be targeted by both guide RNAs confirming the absence of predicted offtargets. Indeed, as a nickase version of the Cas9 enzyme was used for the selection of TERRA-MS2 clones, a perfect match of two different guide RNAs at a specific distance from each other is required in order to promote integration of the exogenous DNA sequence (MS2-cassette) (References 32 and 33 in the main text).

## Slide 2
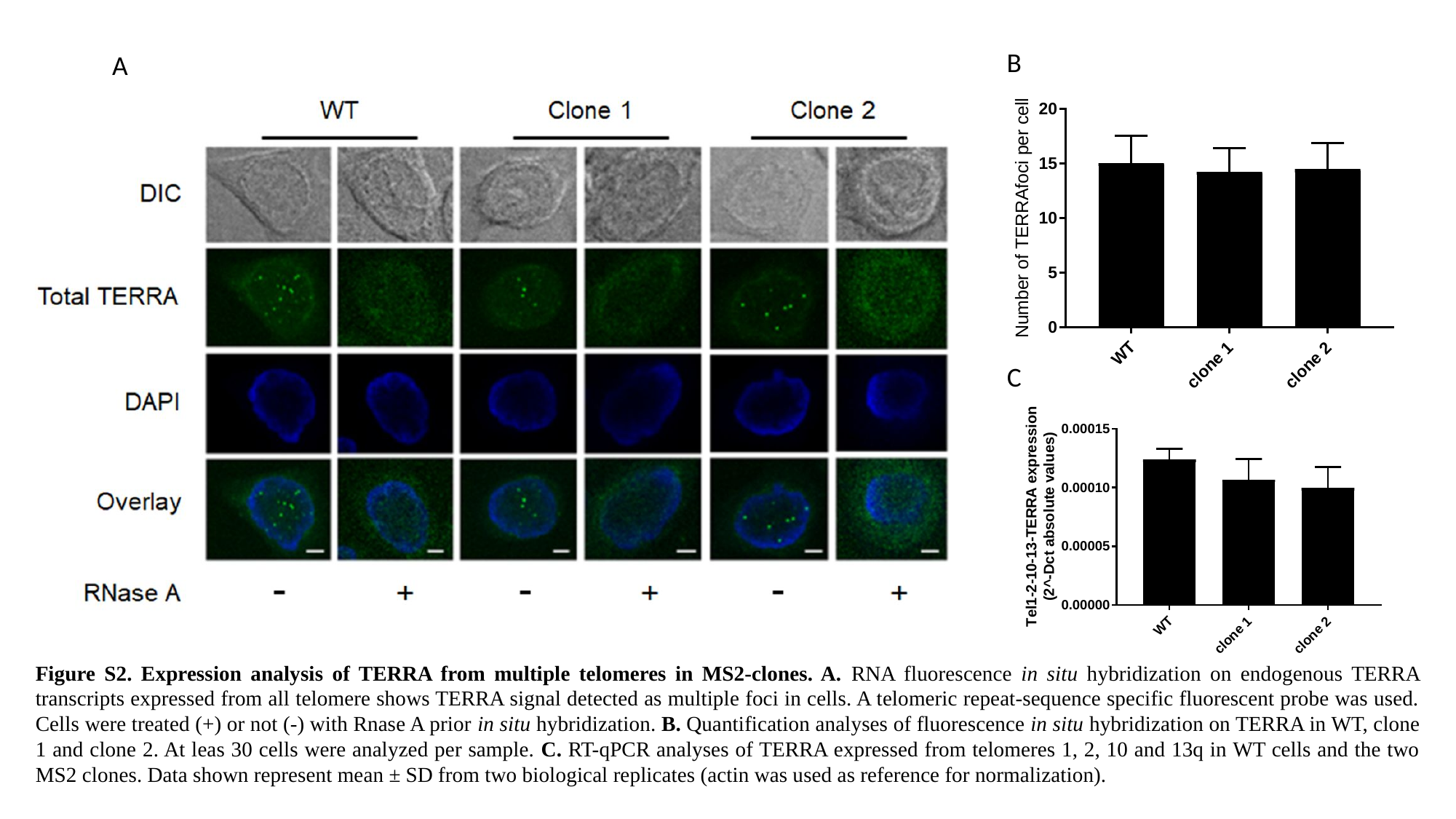

B
A
C
Figure S2. Expression analysis of TERRA from multiple telomeres in MS2-clones. A. RNA fluorescence in situ hybridization on endogenous TERRA transcripts expressed from all telomere shows TERRA signal detected as multiple foci in cells. A telomeric repeat-sequence specific fluorescent probe was used. Cells were treated (+) or not (-) with Rnase A prior in situ hybridization. B. Quantification analyses of fluorescence in situ hybridization on TERRA in WT, clone 1 and clone 2. At leas 30 cells were analyzed per sample. C. RT-qPCR analyses of TERRA expressed from telomeres 1, 2, 10 and 13q in WT cells and the two MS2 clones. Data shown represent mean ± SD from two biological replicates (actin was used as reference for normalization).

## Slide 3
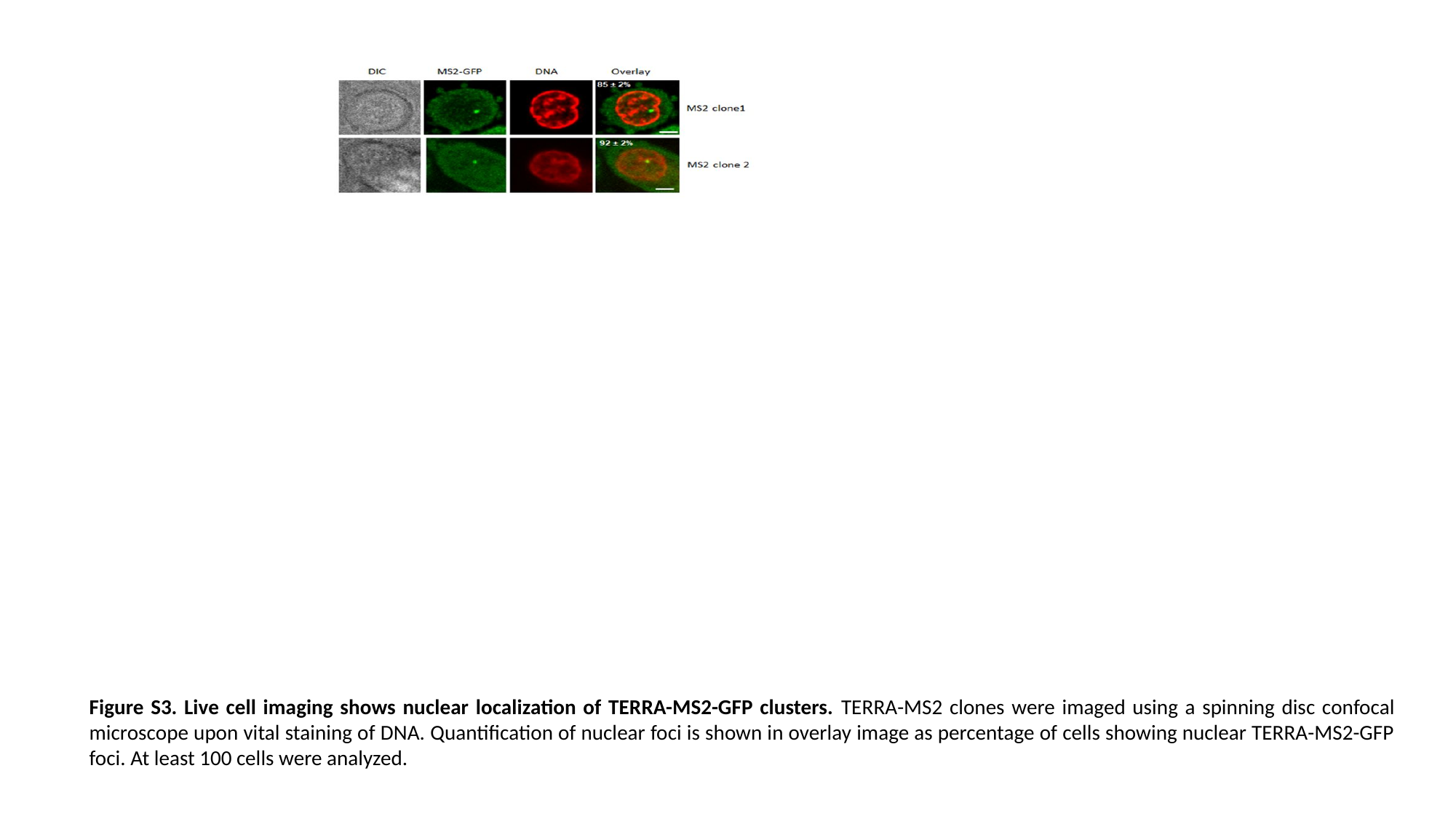

Figure S3. Live cell imaging shows nuclear localization of TERRA-MS2-GFP clusters. TERRA-MS2 clones were imaged using a spinning disc confocal microscope upon vital staining of DNA. Quantification of nuclear foci is shown in overlay image as percentage of cells showing nuclear TERRA-MS2-GFP foci. At least 100 cells were analyzed.

## Slide 4
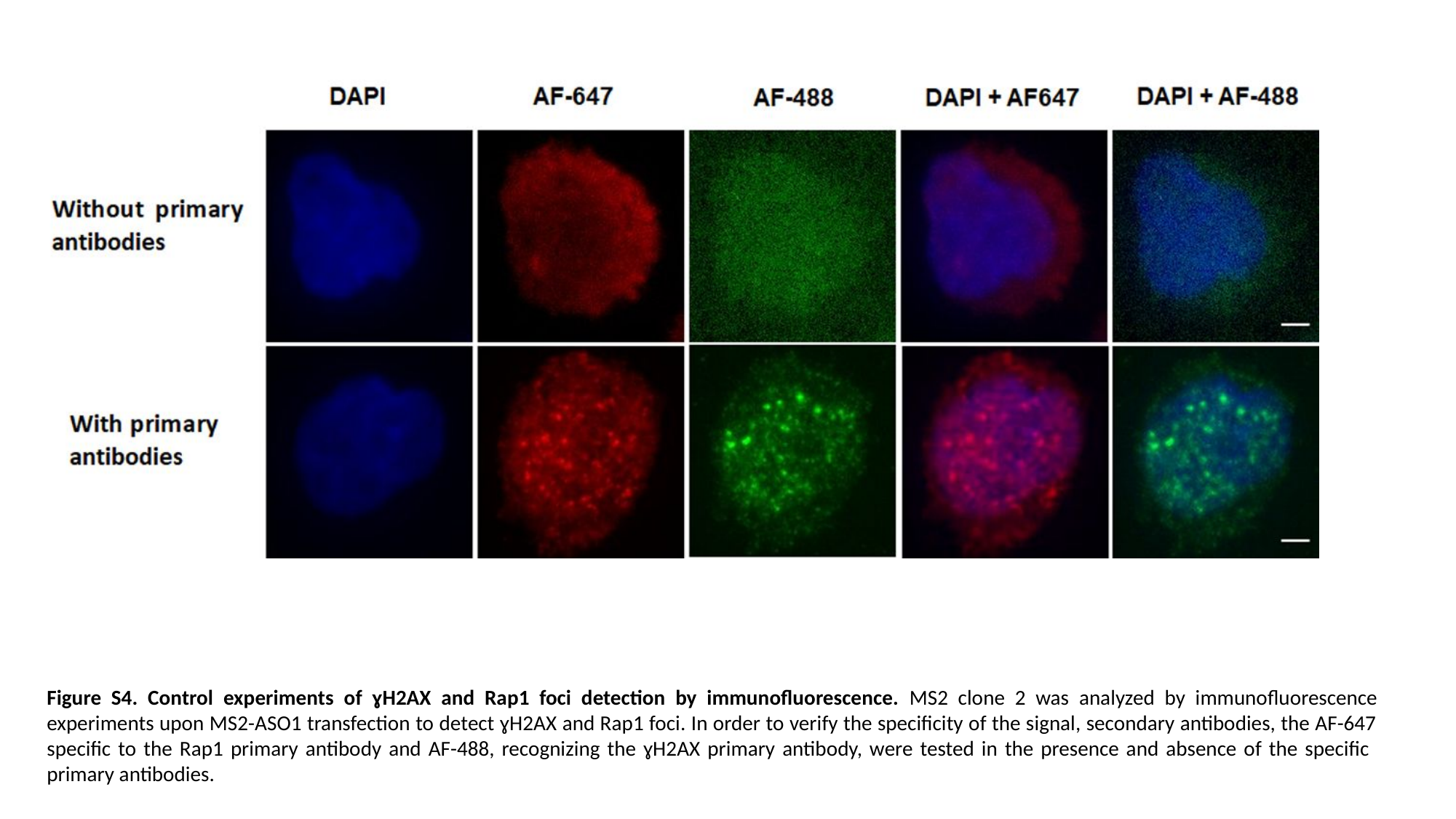

Figure S4. Control experiments of ɣH2AX and Rap1 foci detection by immunofluorescence. MS2 clone 2 was analyzed by immunofluorescence experiments upon MS2-ASO1 transfection to detect ɣH2AX and Rap1 foci. In order to verify the specificity of the signal, secondary antibodies, the AF-647 specific to the Rap1 primary antibody and AF-488, recognizing the ɣH2AX primary antibody, were tested in the presence and absence of the specific primary antibodies.

## Slide 5
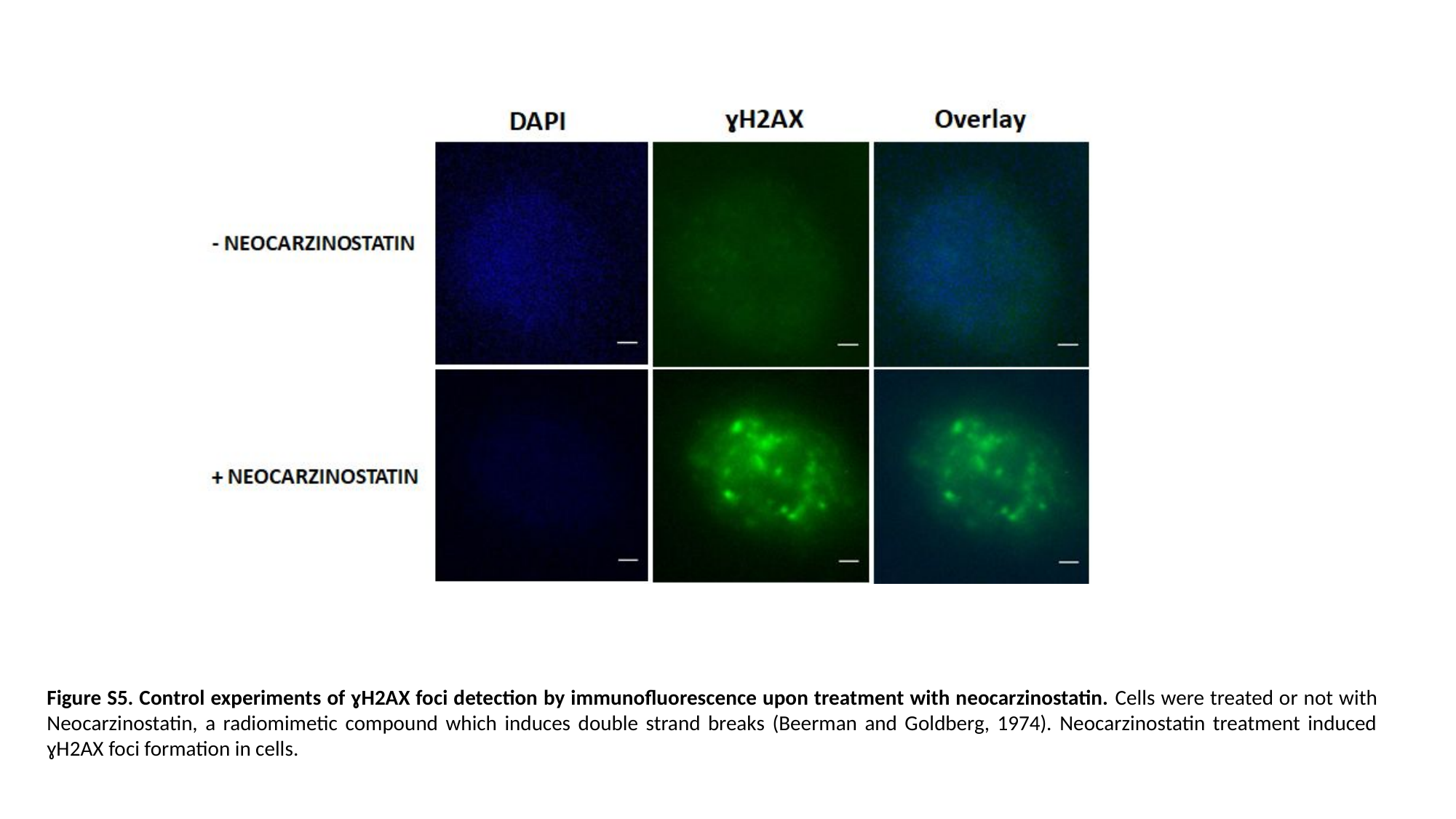

Figure S5. Control experiments of ɣH2AX foci detection by immunofluorescence upon treatment with neocarzinostatin. Cells were treated or not with Neocarzinostatin, a radiomimetic compound which induces double strand breaks (Beerman and Goldberg, 1974). Neocarzinostatin treatment induced ɣH2AX foci formation in cells.

## Slide 6
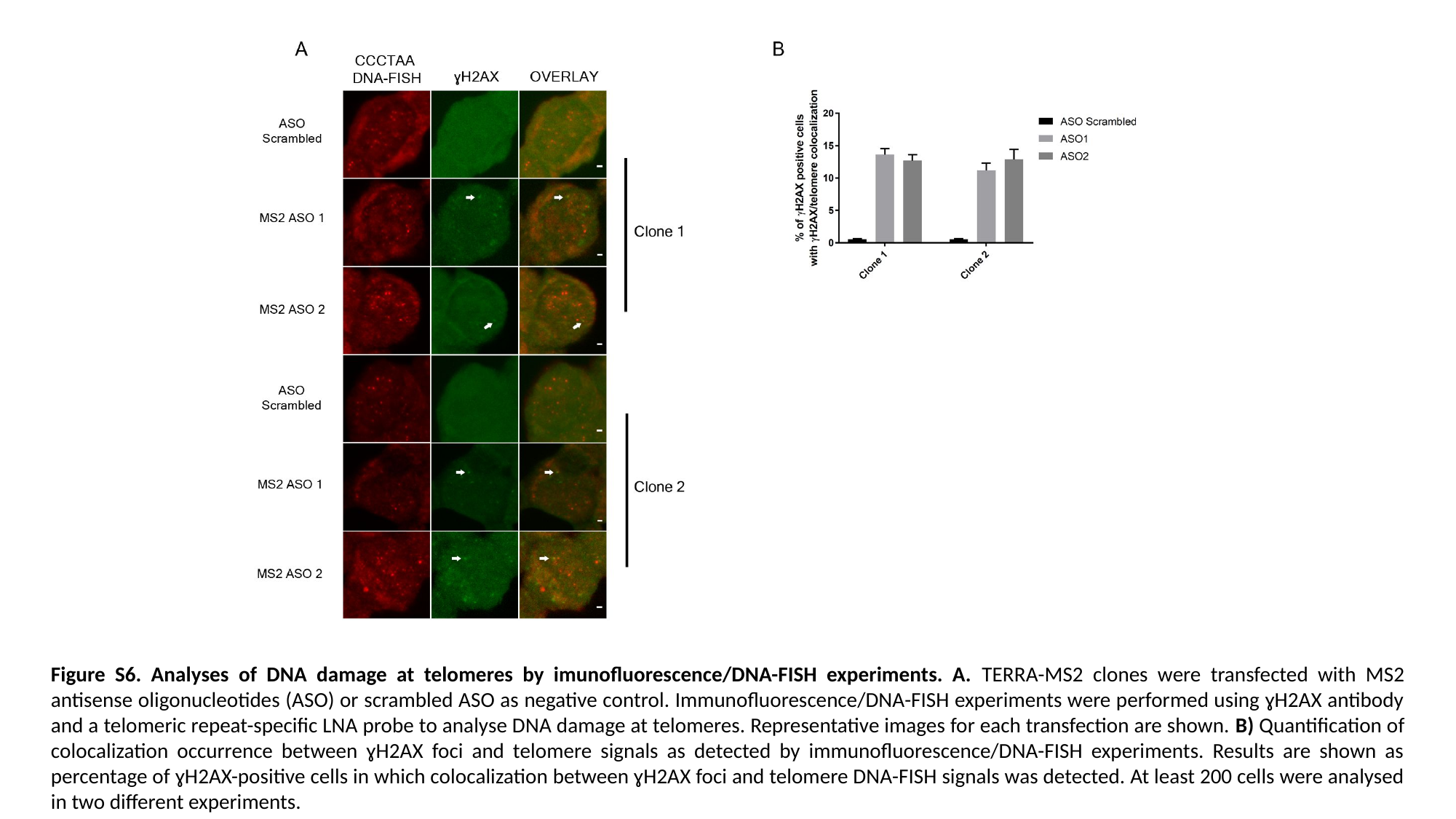

Figure S6. Analyses of DNA damage at telomeres by imunofluorescence/DNA-FISH experiments. A. TERRA-MS2 clones were transfected with MS2 antisense oligonucleotides (ASO) or scrambled ASO as negative control. Immunofluorescence/DNA-FISH experiments were performed using ɣH2AX antibody and a telomeric repeat-specific LNA probe to analyse DNA damage at telomeres. Representative images for each transfection are shown. B) Quantification of colocalization occurrence between ɣH2AX foci and telomere signals as detected by immunofluorescence/DNA-FISH experiments. Results are shown as percentage of ɣH2AX-positive cells in which colocalization between ɣH2AX foci and telomere DNA-FISH signals was detected. At least 200 cells were analysed in two different experiments.

## Slide 7
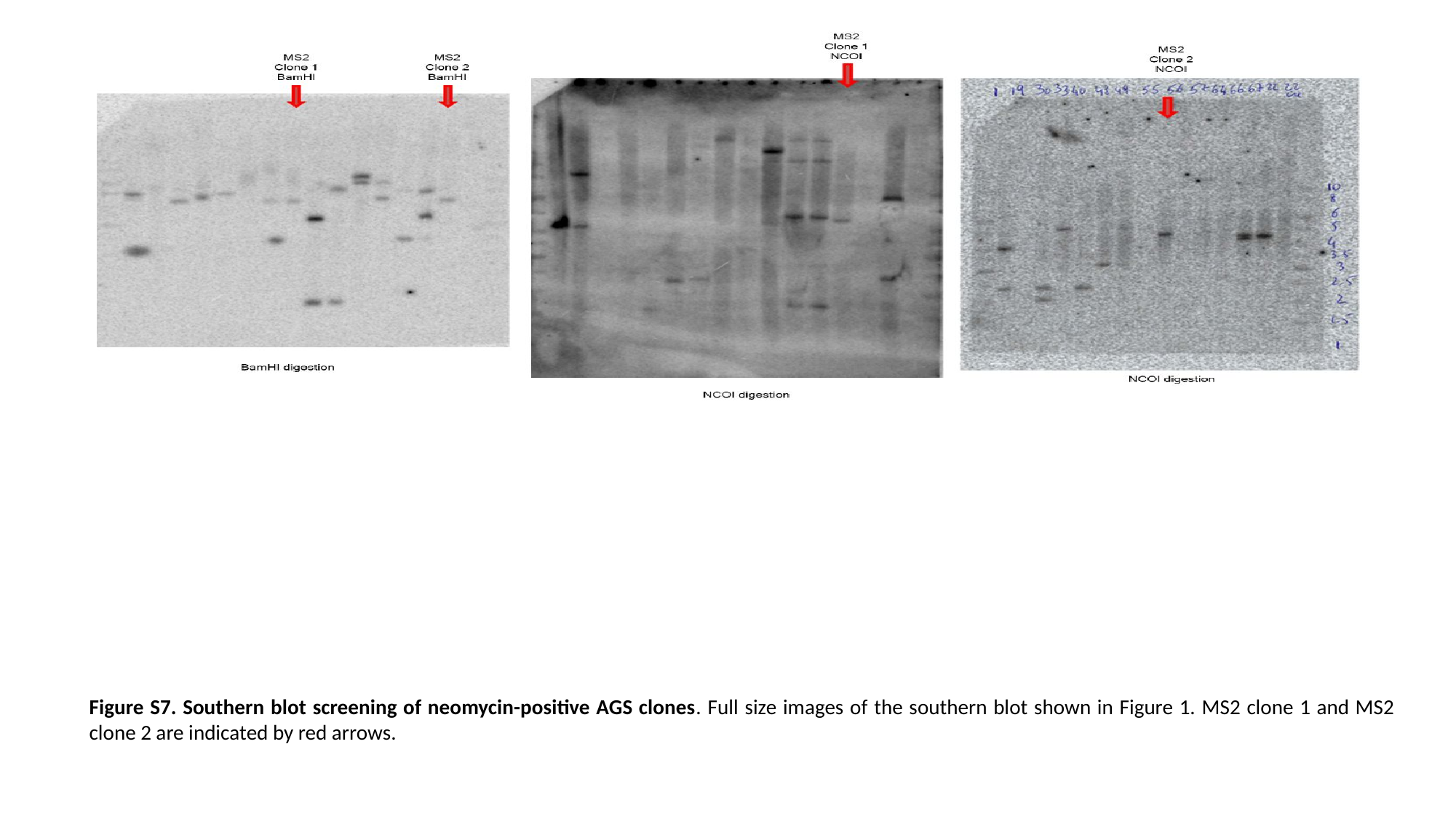

#
Figure S7. Southern blot screening of neomycin-positive AGS clones. Full size images of the southern blot shown in Figure 1. MS2 clone 1 and MS2 clone 2 are indicated by red arrows.

## Slide 8
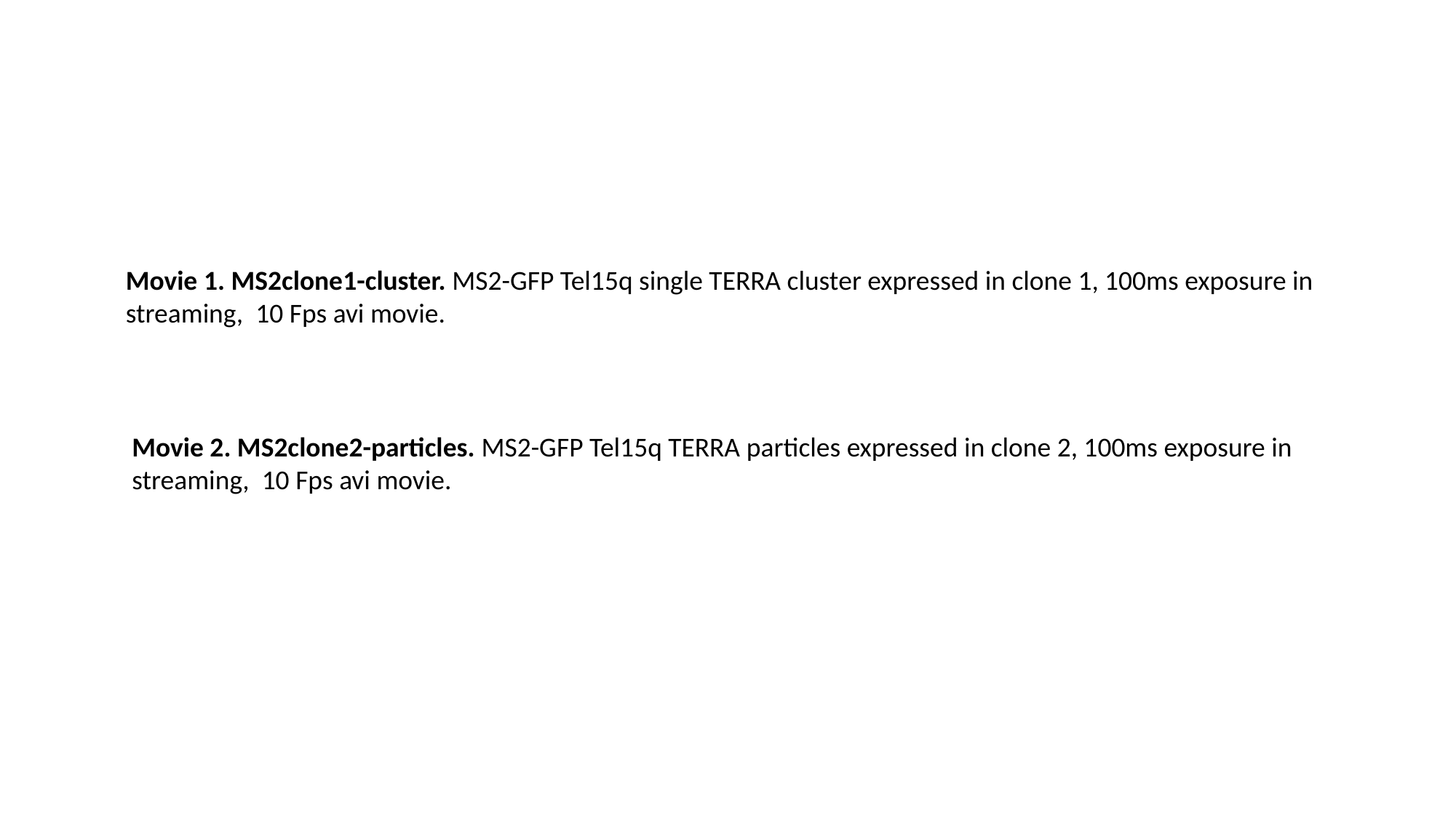

Movie 1. MS2clone1-cluster. MS2-GFP Tel15q single TERRA cluster expressed in clone 1, 100ms exposure in streaming,  10 Fps avi movie.
Movie 2. MS2clone2-particles. MS2-GFP Tel15q TERRA particles expressed in clone 2, 100ms exposure in streaming,  10 Fps avi movie.
